# The connectivity network underlying the German’s Twittersphere: a testbed for investigating information spreading phenomena

**DOI:** 10.1038/s41598-022-07961-3

**Published:** 2022-03-08

**Authors:** Daniel Thilo Schroeder, Johannes Langguth, Luk Burchard, Konstantin Pogorelov, Pedro G. Lind

**Affiliations:** 1 Simula Research Laboratory, High Performance Computing, 1364 Fornebu, Norway; 2 Technical University of Berlin, Distributed and Operating Systems, 10623 Berlin, Germany; 3 Department of Computer Science, Oslo Metropolitan University, P.O. Box 4, St Olavs plass, 0167 Oslo, Norway; 4 Artificial Intelligent OsloMet Lab, Pilestredet 52, 0130 Oslo, Norway; 5 NordSTAR-Nordic Center for Sustainable and Trustworthy Artificial Intelligent, Pilestredet 52, 0166 Oslo, Norway

**Keywords:** Computer science, Information technology, Statistics

## Abstract

Online social networks are ubiquitous, have billions of users, and produce large amounts of data. While platforms like Reddit are based on a forum-like organization where users gather around topics, Facebook and Twitter implement a concept in which individuals represent the primary entity of interest. This makes them natural testbeds for exploring individual behavior in large social networks. Underlying these individual-based platforms is a network whose “friend” or “follower” edges are of binary nature only and therefore do not necessarily reflect the level of acquaintance between pairs of users. In this paper,we present the network of acquaintance “strengths” underlying the German Twittersphere. To that end, we make use of the full non-verbal information contained in tweet–retweet actions to uncover the graph of social acquaintances among users, beyond pure binary edges. The social connectivity between pairs of users is weighted by keeping track of the frequency of shared content and the time elapsed between publication and sharing. Moreover, we also present a preliminary topological analysis of the German Twitter network. Finally, making the data describing the weighted German Twitter network of acquaintances, we discuss how to apply this framework as a ground basis for investigating spreading phenomena of particular contents.

## Introduction

Over the past two decades, online social networks (OSN) have become a part of the daily life of billions of individuals worldwide. Since their operation inherently generates data, OSNs allow the study of human behavior at massive scales. Simultaneously, advancements in high-performance and cloud computing contributed to the availability of affordable, powerful computer hardware, which makes it possible for researchers to analyze the large datasets generated in OSNs. Consequently, OSNs have been the subject of many academic papers in the past decade. Among the topics is the study of the distribution of information on a large scale^1^.

Information distribution can be modeled by considering individuals as stateful entities frequently exposed to information accumulated and processed to form opinions, which again serve as new input^2^. The connections through which the exchange of information and thus individuals’ influence occur are in many cases part of the core functionality of an OSN and are often called *friend*- or *follower* relationships. However, these connections are unweighted and thus signify only the presence of interest. They do not reflect how individuals actually interact or influence each other. Uncovering the underlying network of acquaintances of the German Twittersphere would provide a testbed for further investigations and open the possibility for further investigation of dynamical processes, such as the propagation of information—and misinformation—on a network of acquaintances. Therefore, there is a need for obtaining weighted relationships from the communication that occurs between individuals.

The main aim of this paper is to uncover the network of (weighted) acquaintances of the German Twitter platform, in order to assess the dynamical aspects on tweet sharing activity. To this end, we introduce a framework to generate large-scale networks of social connectivity built up using Twitter interaction data only. We archive this using a weighting function that measures the strength of connectivity for Twitter user pairs based on their communication and sharing behavior. In particular, we define connectivity based on the reaction time and the number of exchanged retweets. Since using Twitter’s follower mechanism is an unweighted expression of interest that reflects how individuals have chosen to relate to each other rather than how they actually interact with each other, and because it is biased by e.g. Twitter’s recommender algorithm^3^, its onboarding process, and real-world encounters^4^, we argue that the networks resulting from the proposed method make a better testbed for studying information diffusion than Twitter’s underlying follower network. Finally, we apply the proposed method to derive the network underlying significant parts of the German Twittersphere and present a systematic structural analysis of the derived network using standard tools^5^ and topological properties from network analysis. While not addressing any specific problem in the field of information diffusion or social network dynamics directly, we contribute by delivering the foundation for doing so. However, due to its sheer size, with more than 30 million nodes and 120 million edges, we are limited by hardware, layouting- and rendering algorithms and only capable of displaying an excerpt. The datasets encoding the German Twitter network of acquaintances is available at https://datasets.simula.no/ExE1Dlex4PyE78q9BXFv/.

There have been several proposals, several cited in the manuscript, to define and study networks based on online social networks. Similar approaches, due for a much smaller dataset, were already proposed e.g. for the South African Twitter, with a network-based approach to map the main constituencies discussing specific topics on Twitter^6^. In Ref.^7^, the authors examine with whom german political journalists interact on Twitter and what information they share. They use a hybrid approach combining a content analysis and a network analysis. However, the observed population is very small, focused in the twitter “subgroup” of journalists and data is not available for further investigations. In^8^, the interactions of 150 Dutch journalists and politicians is addressed. The authors claim that acquaintance networks from Twitter have an underlying structure that is more detailed than one would expect from the list of followers, based in simple cluster analysis. Additionally, the same claim has been supported in a work^9^ analysing around 3.2 million users with the aim of building an interaction network to map communities of acquaintances.

Compared to other OSNs, Twitter offers the possibility to examine diverse communication forms in a more differentiated manner. Twitter supports retweets, i.e., sharing others’ content without commenting on it. Hereupon, the shared content or retweet is openly accessible, and those who follow the sharing user receive the shared content. Naturally, frequent resharing of content indicates a strong interaction between individuals. However, the speed at with content is shared also represents an important dimension. Thus, understanding the frequency and reaction time with which pairs of individuals share such retweets as a measure of interaction intensity and influence seems appropriate.

Substantial efforts have been made in this scope. For instance, Granovetter’s well-known work “The Strength of Weak Ties” distinguished between the strong and weak ties of an individual and concluded that, as the name suggests, weak ties are important^10^. Implications of the strength of the connections are numerous far-reaching^11–13^, and well understood. However, an implication that is not immediately obvious is the role of *information content* when considering the communication in user pairs. This content is strongly correlated with the strength of the ties binding pairs of persons. People who tend to communicate over strong ties tend to know each other very well and therefore need less complex protocols and less communication accuracy in order to express themselves^14^. In other words, since there is a greater common prior knowledge, information needs less context to be understood.

Communication “short-cuts” also promote communication skills and intelligence. For instance, it has been reported^15^ that people with weaker ties, who need to explain complex content, must use more elaborate language skills and strategies. Thus, it is of utmost importance to assess the strength of ties that bind pairs of individuals interchanging information.

Social networks are also influenced by location. It is more likely to be a friend to somebody who is geographically close^16^ than to somebody who has his or her center of life at a great distance. Toole et al.^17^ used mobile geo data to show that not just the probability of friendship is predictable but that making use of the correlation between tie strength and mobility similarity allows for classifying social relationships.

While there are numerous examples of networks we have to deal with, daily acquaintance networks have topological features that differ fundamentally. Being single-scale and small world networks are probably the most important. However, González et al.^18^ were able to show that networks with similar topological properties can be created from agent simulations. In contrast to the approach presented in this work, here, a collision between two simulated agents moving in a square-shaped cell leads to adding a new edge to the network.

In Twitter networks, link prediction is commonly referred to as the problem of predicting the existence of a follower connection between two users. Tsugawa et al.^19^ introduced a method that, additionally to already established network-based link prediction methods like Common Neighbours^20^ or Resource Allocation^21^, use user activity and retweeting in particular, to gain further improvement. There are important differences compared to our work , such as the proposed concept of RetweetViews. Despite having apparent similarities with the scoring proposed below, this measure differs substantially in the way that it considers transitive retweets. Here, the number of tweets that are authored by a user *i* and retweeted by any of its followers *k* that follow a user *j* is used as a measure that contributes to the link prediction.

Similar to Tsugawa et al., Ahmed et al.^19^ introduced a more generic link prediction based on the FriendTNS^22^ method. Like in Tsugawa et al., the algorithm is supplemented with interaction data and benchmarked on the Twitter2.9k dataset. Here, like in the previous suggestion, no reaction time is considered. Furthermore, the Twitter2.9k dataset for benchmarking is rather small compared to the dataset used in our work. Again, as the focus lies on the prediction of links (follower relations on Twitter), there is no analysis of topological properties.

We begin by presenting the dataset used to build the network and continue with the methodology. Then, we introduce the weighting function used to measure the interaction between pairs of users and present the properties resulting from applying it to all pairs of users in the dataset. Using these set of tools we then describes the building process for the entire network and discuss it in some detail. Features of the German Twitter network of acquaintances is discussed separately in what concerns first and second neighborhoods. Finally, in the discussion and conclusions we propose some future directions which could be done to use the outcome of this paper to investigating the spreading of information and misinformation in social networks.

**Table 1:** Twitter lists including the initial accounts from which the data was collected.

Twitter list	
kunigkeit/pod-parteien-politiker	wahl_beobachter/mdl-sachsen-anhalt
VeHoltz/bundespolitik	wahl_beobachter/mdl-baden-w-rttemberg
MarcusSchwarze/fakes	wahl_beobachter/mdl-nrw
wahl_beobachter/botschaften	wahl_beobachter/mdl-saarland
wahl_beobachter/kandidaten-europawahl	wahl_beobachter/mdl-schleswig-holstein
wahl_beobachter/bundesministerien	wahl_beobachter/mdl-mecklenburg-vorpommen
wahl_beobachter/bundestagsfraktionen	wahl_beobachter/abgeordnetenhaus-agh
wahl_beobachter/mdb-bundestag	wahl_beobachter/bundesregierung
wahl_beobachter/mdl-bayern	wahl_beobachter/politikwissenschaftler
wahl_beobachter/mdl-hessen	wahl_beobachter/ministeriums-twitterati
wahl_beobachter/mdl-niedersachsen	wahl_beobachter/alle-25-parteien-ep2014
wahl_beobachter/mdbb-bremen	wahl_beobachter/deutsche-mep-2019-2024
wahl_beobachter/mdl-brandenburg	wahl_beobachter/open-government-hamburg
wahl_beobachter/mdl-sachsen	wahl_beobachter/mdhb-hamburg
wahl_beobachter/mdl-rheinland-pfalz1	AfD/verifizierte-accounts
wahl_beobachter/mdl-th-ringen	AfD/bundestagsabgeordnete

## Collecting and processing data from the German Twitter network

Since Twitter’s Terms of Service prohibit storing large datasets, we choose a streaming-based approach storing metadata only. We keep only in-stream-anonymized user Ids, and the corresponding timestamps to create the tweet-retweet-user mapping. Moreover, we do neither store or process any other information. The data collection took place using a custom build framework for Twitter graph analysis^23^, and a custom scraping strategy. The latter is required since we target to fetch massive amounts of data that are as *dense* as possible. Here, dense refers to a comprehensive historical sample of tweets for each user. Furthermore, we target users that are likely to interact with each other. Twitter’s API does not support to fetch data in the required way, therefore we used our custom approach^24,25^ which, contrary to the widely used Search API, analyzes individual users’ neighborhoods.

The data collection started in late 2019 and stretched to mid-2020. During this time, we analyzed more than one billion historical statuses from user timelines. Among them are 400 million tweets and 300 hundred million retweets. All statuses in total are written or shared by about 30 million users. The goal was to collect and analyze as much tweet and retweet metadata as possible.

Based on a set of 2638 Twitter accounts of German personalities close to politics that we derive from the Twitter lists in Table 1, we first fetch a user’s most recent 3,200 tweets and later their first and second neighborhoods in the follower network. Subsequently, we analyze the most recent tweets of the newly acquired users, ignoring those that are not written either in German or in English. We repeated this process iteratively with users only tweeting in German until the number of newly added users decreased significantly.

One should note that the Twitter API only allows limited access to user data. Twitter’s limit of only 3200 tweets that are accessible for each user timeline implies that only a small portion of the tweets authored by highly active users contribute to our dataset. Moreover, Twitter allows its users to set their profiles to private, i.e. allowing only direct followers to access that user’s content. For that reason, data from private profiles is excluded from our consideration. Note that the work presented here examines the interaction that arises from the sharing of content. Users who have never retweeted another user are therefore not considered.

## Building the interaction network underlying the Twitter database

Composing interaction networks and examining their topological properties relies on the evaluation of Twitter data. Therefore, we switch to the corresponding terminology for the remainder of this paper. Twitter reflects an individual’s characteristics such as length of membership, name, and place of origin in the form of a user account. Accordingly, we call the individuals users. Users communicate via short messages or tweets of up to 280 characters that are accessible to the public. Interaction takes place by commenting on tweets or sharing them. A shared tweet is called a retweet and can be commented on when shared. In the latter scenario, the retweet is then called a quoted-retweet.

### Assessing the intensity of pairwise interactions for information exchange

To assess the strength of connections between pairs of users, we derive two main properties from the Twitter dataset, namely, we define an average reaction time for a retweet and a so-called “tweeting rate”. In this way, we postulate that the number of retweets and the reaction time with which two users exchange information are the fundamental properties for describing their connectivity.

To measure the retweeting rate, we introduce the quantity

$$\begin{aligned} P_{ij} {:}{=}\hbox {set of tweets authored by} \,i \,\hbox {and shared by} \,j. \end{aligned}$$

The retweeting rate at which user *j* retweets user *i* is given by $1/n_{ij}$, where $n_{ij}=|P_{ij}|$ is the cardinality of $P_{ij}$.

**Figure 1:**
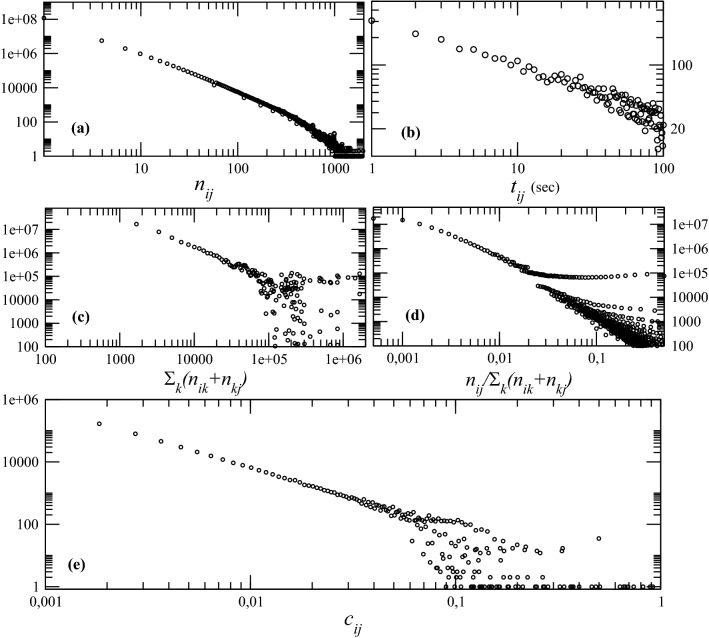
(**a**) Distribution of the number of tweets $n_{ij}$ that a user *j* retweets from another user *i*. (**b**) Distribution of the time-span $t_{ij}$ between the instant user *i* tweeted a tweet and user *j* retweeted it. (**c**) Distribution of the sum in Eq. (3) for each pair of users. (**d**) Distribution of the part in Eq. (3) depending only on $n_{ij}$. (**e**) Distribution of the values of the weights $c_{ij}$ as defined in Eq. (3).

In Fig. 1a we see the distribution of $P_{ij}$ from the Twitter dataset. From this plot, it is clear that for the majority of user-pairs, there is either a rare exchange of information or no exchange at all. While there are other forms of interaction, such as private messages, comments, or quoted-retweets, we define the weighting function exclusively in accordance with retweets. We argue that this restriction is reasonable due to the ”purity” of retweets, i.e., the lack of opportunity to comment on the shared content. Retweets implicitly prohibit the negation of the initial statement and can thus imply agreement^26–28^. Admittedly, this statement is not universal because situation-, users- or target group-properties indirectly provide context, but the coherence results from a retweet’s nature and seems, as such, conceivable.

To measure the reaction time, we first introduce the timestamp at which user *i* publishes a tweet *m*, represented henceforth as $t_{i}(m)$, and the time at which user *j* shares *i*’s given tweet, represented as $t_{j}(m)$. Thus, the reaction time of user *j* to *i*’s tweet *m* is the time difference between a tweet *m* authored by *i* and shared by *j* is given as

1 $$\begin{aligned} t_{ij}(m) {:}{=}t_{j}(m)-t_{i}(m) \, . \end{aligned}$$

This time interval is used to define the average reaction time over all tweets that were shared between *i* and *j*

2 $$\begin{aligned} T_{ij} {:}{=}\frac{1}{n_{ij}} \sum _{m = 1}^{n_{ij}} {t_{ij}(m)} \, . \end{aligned}$$

In Fig. 1b, we show the distribution of the reaction differences over all tweet–retweet pairs $t_{ij}(m)$ in seconds, for the entire dataset. The average reaction for retweeting happens typically within the first seconds. Moreover, similarly to $n_{ij}$, $t_{ij}(m)$ seems to follow approximately a power-law.

We claim that, up to some extent, the reaction time reflects the level of connectivity the retweeter (user *j*) has with respect to the tweet author (user *i*). Indeed, we assume that the average reaction time implicitly represents a gauge of activity. Users who are more active react more rapidly to each other’s content. Moreover, by the very nature of things, someone who approves the same attitude and is particularly interested in someone else’s content will not hesitate or need to be convinced and, thus, tends to react instantly. Furthermore, Twitter offers the option to follow users, i.e., if user *j* follows user *i*, user *j* receives *i*’s tweets exclusively via his/her newsfeed, allowing active users to react instantly. User *j* activating Twitter’s build-in notification feature can even extend the following mechanism. For every of *i*’s tweets, *j* then receives not only a newsfeed update but also a push message, allowing *j* to react even more rapidly. It is important to note that a follower relation does not necessary imply interaction and rapid interaction in particular. It is entirely possible to follow but never retweet.

Suppose the connection between two users is consistent over the entire history of their interactions, characterized by short reaction times, i.e., the willingness to share the other’s statement. If a user consistently responds quickly to another’s messages by sharing them without commenting, that deem that user to be particularly active. In this scope, we introduce the term *connectivity* to describe the stronger or weaker tendency to share Twitter content. Having defined both the retweeting rate and the reaction time, we can now introduce a property which measures the connectivity strength between two users, *i* and *j*, namely

3 $$\begin{aligned} c_{ij}(t) {:}{=}\frac{n_{ij}}{\sum \limits _{k = 1}^{N}{(n_{ik} + n_{kj})}} \frac{1}{T_{ij}}. \end{aligned}$$

The weight $c_{ij}$ accounts for the number of tweets that user *j* shared from user *i* with the corresponding reaction time, and it increases inversely to the frequency and reaction time. Notice that in the denominator of Eq. (3) there are two sums, normalizing the number $n_{ij}$ of tweets authored by a user *i* and shared by another *j*. One sum, $\sum _{k = 1}^{N} {n_{ik}}$, is the number of tweets published by *i* and shared by any user, representing a sort of popularity of user *i*. The other sum, $\sum _{k = 1}^{N} {n_{kj}}$, is the number of tweets published by any user and shared by *j*, representing a sort of willingness to share content of user *j*. In this way, the connectivity weight $c_{ij}$ is based on the assumption that (1) it increases with the total number $n_{ij}$ of tweets from user *i* to user *j*, normalize by the additive effect of *i*’ popularity and *j*’s willingness to share, and (2) it decreases with the average reaction time $T_{ij}$, i.e. the longer user *j* takes to retweet user *i* the weaker their connectivity is. Figure 1c shows the distribution of the both sums together for each pair of users, while Fig. 1d shows the distribution of the normalized number of tweets between pairs of users, $n_{ij}/\sum _{k}(n_{ik}+n_{kj})$. For large values of the normalized amount of retweets, one observes parallel (almost) constant frequencies. This is a numerical effect from very high heterogeneities in the network, namely due to a few very popular users with very high number of retweets—the $n_{ik}$ in the sum in Eq. (3)—present in the normalization sum for almost any pair of users, we observe very similar (large) values of the normalizatio sum for a broader range of values of $n_{ij}$.

In Fig. 1e, we show the distribution of the weights $c_{ij}$ which seems to also follow approximately a power-law. Notice that sharing a tweet is a “directional” activity: a fan of a pop-star can have a very strong interaction with his or her idol, while, on the other hand, the pop-star has a weak connection with his or her fan. The definition in Eq. (3) is prone to some noise, since many factors can influence both the number of retweets and the average time between retweets shared by the same pair of users, some of them characteristic of the two individuals. However, we argue that, from the fundamental concept of interaction strength, it is natural to assume it being influenced not only by the (normalized) amount of tweets shared between pairs of users but also by some time rate in which these retweets happens, which in our definition is tuned by $1/T_{ij}$. Otherwise, interacting with a fixed amount of tweets in 1 h and 1 week would be accounted with the same interaction strength.

### Beyond Twitter’s follower-network: building an empirical social network with weighted interactions

Based on the dataset presented in “Building the interaction network underlying the Twitter database” and on the weighted function introduced in "Assessing the intensity of pairwise interactions for information exchange", we derive a network underlying the Twitter social network, as sketched in the dataset repository https://bit.ly/3fIRHpu.

**Figure 2:**
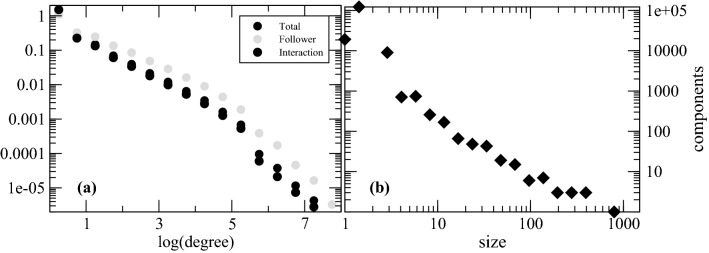
(**a**) Comparison between the degree distribution of the main connected component of the derived network from the Twitter dataset and the one of the known network of followers. The existence of an edge in the Twitter follower network is associated with the $c_{ij}$-score $>0$ of the edge in our derived weighted network. (**b**) Size distribution of the connected components not included in the main component. All these components have sizes not larger than 1000 nodes, which justifies neglecting these components and focusing the topological analysis on the main component having approximately thirty million nodes (see text).

We performed a connected component analysis, which reveals a major component with a size of about 30 million nodes and 120 million edges. Separated from this main component, there are about 150 thousand components overall. As illustrated in Fig. 2b, about 400 of these smaller isolated “islands” have more than 10 nodes, 24 containing more than 100 nodes, and just two including around 1000 nodes. Therefore, a component isolated from the main component is indeed isolated, i.e., users have not shared content with users outside the component, nor has their content been shared by users outside the component. Henceforth, we only consider the main connected component, filtering out all other smaller components.

The connected component analysis was performed considering each user as part of a component, regardless of his or her activity or impact, since the goal of the connected component analysis is to analyse the fragmentation of the overall discourse. In other words, there are two possible “extreme cases” of users: (1) passive users who share content but do not distribute their own content and (2) users who distribute content but never share it. For the connected component analysis, both of the aforementioned user groups are included.

The purpose of this work is to provide a framework that allows for large-scale modelling of dynamic processes solely based on Twitter’s interaction data. To that end, we now compare our derived network with the follower network accessible through Twitter’s API. Twitter gives its users the ability to follow any other user, meaning to subscribe to his/her content and, moreover, to receive notifications if requested. The content written and shared by those who are followed appears in the subscriber’s newsfeed. To understand whether sharing content coincides with the active decision to subscribe to another user’s content, we checked for each directed edge with $c_{ij} > 0$ whether a corresponding edge exists in Twitter’s follower network. Results are shown in Fig. 2a and indicate that the existence of edges obtained with the proposed approach often coincides with the existence of follower connections on Twitter.

This result is not surprising because users who interact with each other by retweeting tend to decide to follow each other. However, there is also the possibility of sharing content from accounts that one does not follow, for example, via third-party websites; even so, this seems not to happen frequently. Twitter’s notification and recommender algorithms also presumably contribute to the fact that content is shared more often by followed users. In addition, Twitter makes suggestions for potential followers based on shared tweets, followers, and follows. If a piece of content is shared by someone who is not followed, it is quite conceivable that a follower edge will be added afterwards, based on this recommendation.

## Topological analysis of the Twitter interaction network

**Figure 3:**
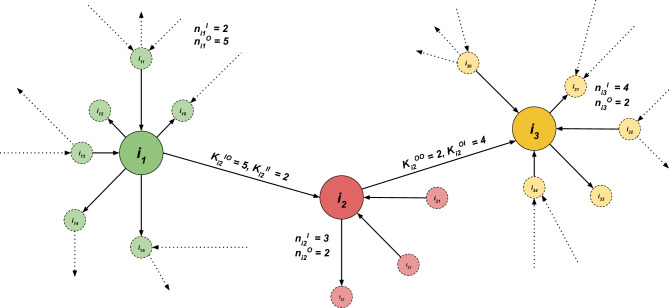
Illustration of topological properties characterizing first and second neighborhoods. The figure does not reflect any structural properties of the derived network itself and is for illustration only. Three adjacent nodes $i_{1}$, $i_{2}$ and $i_{3}$ are shown. The neighborhood of each node is highlighted with the color of the corresponding node, and the node itself is labeled with the respective $n_{i}^{O}$ and $n_{i}^{I}$ (see Eq. 4). It should be noted that the node $i_{2}$ in the center is connected with an outgoing edge to $i_{3}$ and an incoming edge to $i_{1}$. Summing the incoming and outgoing edges of $i_{1}$’s neighbors gives the value for the corresponding $K_{i_{2}}^{II}$ or $K_{i_{2}}^{IO}$ (see Eq. 6). The same applies to the node $i_{3}$ but with the outgoing edge from $i_{2}$. Therefore, we receive $K_{i_{2}}^{OI}$ or $K_{i_{2}}^{OO}$.

In this section, we present a description of the main topological features of the derived network. While the analysis is specific of the German Twitter dataset, it can be straightforwardly extended to other Twitter datasets or similar data. We divide the analysis into three parts. First, we describe the first neighborhood’s topological properties; later, we will investigate the properties characterizing the nodes’ second neighborhoods. A definition for first and second neighbourhood is given below in this section. Finally, we address the entire network’s global measures, such as the average shortest path length and betweenness centrality. The aim is to examine the activity, level of connectivity, and impact of our dataset users. A user’s influence, activity, level of connectivity, and impact is compared to that of its neighbors, so that statements of the form “The more influential a user *i*, the less active are those that *i* influences” can be derived. Figure 3 illustrates the spatial meaning of the properties considered in this section.

### Assessing the first node neighborhood: *“activity”* and *“impact”* of each user.

We first label the set of nodes that have an outgoing edge to node *i* as ${\mathcal {N}}_{i}^{I}$, and the set of nodes have an incoming edge from node *i* as ${\mathcal {N}}_{i}^{O}$. The total set of nodes, either in one way or the other, is then given by ${\mathcal {N}}_{i}^{A}={\mathcal {N}}_{i}^{I}\cup {\mathcal {N}}_{i}^{O}$. The number of nodes in ${\mathcal {N}}_{i}^{I}$, ${\mathcal {N}}_{i}^{O}$ and ${\mathcal {N}}_{i}^{A}$, which we call in-degree, out-degree, and degree, respectively, are given by

4a $$\begin{aligned} n_{i}^{I}&{:}{=}&\vert {\mathcal {N}}_{i}^{I} \vert \, , \end{aligned}$$

4b $$\begin{aligned} n_{i}^{O}&{:}{=}&\vert {\mathcal {N}}_{i}^{O} \vert \, , \end{aligned}$$

4c $$\begin{aligned} n_{i}^{A}&{:}{=}&\vert {\mathcal {N}}_{i}^{I} \cup {\mathcal {N}}_{i}^{O} \vert \, . \end{aligned}$$

In order to clarify the interpretation, we name the degrees as follows:

$$\begin{aligned} n_{i}^{I} {:}{=}\hbox {size of} \,i'\,\hbox {s influenc}{} \mathbf{ing}\,\, \hbox {neighbourhood} \end{aligned}$$

and

$$\begin{aligned} n_{i}^{O} {:}{=}\hbox {size of} \,i'\,\hbox {s influenc}{} \mathbf{ed} \,\,\hbox {neighbourhood} \, . \end{aligned}$$

Influencing in this context only points to the number of different users that *i* retweeted during its entire lifetime (record in the examined dataset). Another way to put it is that the tie strength $c_{ij}$ is not taken into account. We want to indicate that this function is also helpful as a measure of opinion diversity. Users who share the content of many other users tend to form opinions based on this diversity and are therefore more robust towards content without truth. However, this is only valid with restrictions. The nature of our study does not allow a judgment on so-called filter bubbles^29^ or echo chambers^30^. If a user shares many users’ content, but all those from whom the content is shared are only linked to each other, $n_{i}^{I}$ is not a suitable indicator for the diversity of opinions.

The same applies to the influenced neighborhood $n_{i}^{O}$ which is the number of different users that retweeted *i* at least once throughout their entire lifetime, i.e., with respect to all data points in our dataset. Again, the term *influenced* is not a measure of the depth to which *i*’s content diffuses into the social network. Here, all those who share *i*’s content could exist completely isolated from the rest of the network, talking only to themselves.

The weighted counterparts of both these properties ($n_{i}^{O}$, $n_{i}^{I}$), which we represent by $w_{i}^{I}$ and $w_{i}^{O}$, account for the weighted degree respectively for all incoming and outgoing neighbours of *i*, are defined as

5a $$\begin{aligned} w_{i}^{I}&{:}{=}&\sum _{m \in {\mathcal {N}}_{i}^{I}}^{} {c_{mi}} \, , \end{aligned}$$

5b $$\begin{aligned} w_{i}^{O}&{:}{=}&\sum _{m \in {\mathcal {N}}_{i}^{O}}^{} {c_{im}} \, , \end{aligned}$$

5c $$\begin{aligned} w_{i}^{A}&{:}{=}&w_{i}^{I} + w_{i}^{O} \, . \end{aligned}$$

They have an important meaning for the topological analysis, namely:

$$\begin{aligned} w_{i}^{I} {:}{=}\hbox {activity of user }\,{i} \, , \\ w_{i}^{O} {:}{=}\hbox {impact of user}\,{ i} \, . \end{aligned}$$

Our definition of activity in this context does not consider the diversity of the sources. A user who retweets a particular user *k* often and with fast reaction time can be as active as a user who shares content from many users but does so infrequently. If a user is quick at sharing and thus consuming content and shares, moreover, often or from many other users, the user seems active. For this reason, we believe that active is an appropriate term here.

**Figure 4:**
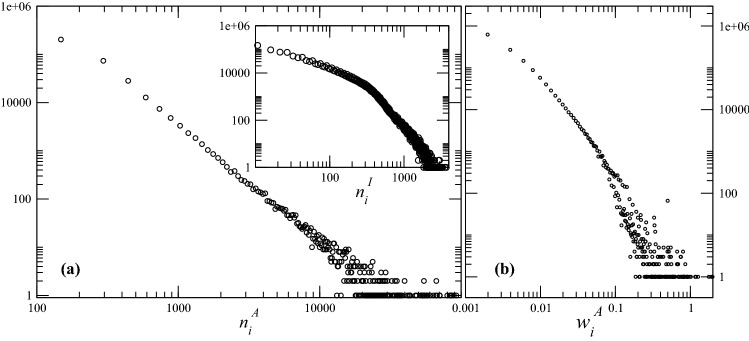
(**a**) Distribution of the total number $n_{i}^{A}$ of neighbors of a node *i*. In the inset we plot the distribution of the size $n_{i}^{I}$ of the influencing neighborhood of each user *i*. (**b**) Distribution of the total weighted degree $w_{i}^{A}$, which sums up the activity and the impact of each user.

Figure 4a shows the distribution of $n_{i}^{A}$ (see Eq. 4c) on a log-log plot. The interpretation of this behavior is that most users neither influence a wide variety of different users, nor are they influenced by a wide variety. The overlay in the same plot depicts the distribution of $n_{i}^{I}$ (see Eq. 4a) also on a log-log scale. Up to $n_{i}^{I} < 130$ the distribution follows $x^{-1}$ and later for $n_{i}^{I} > 130$ $x^{-15/4}$ meaning the number of users influenced by at least 130 users decreases faster than the number of users influenced by less than 130 users. Users *i* with $n_{i}^{I} > 130$ are rare. They are *influenced* by many different users, although this number alone does not imply that they are exposed to diverse opinions. Many such users likely make use of Twitter in a professional or semi-professional capacity.

Figure 4b shows the distribution of $w_{i}^{A}$ (see Eq. 5c). Since $w_{i}^{A}$ is the sum of $w_{i}^{O}$ or a users activity and $w_{i}^{I}$ or a users impact, one observes that up to $w_{i}^{A} < 0.1$, users tend to show a positive correlation between their activity and impact.

**Figure 5:**
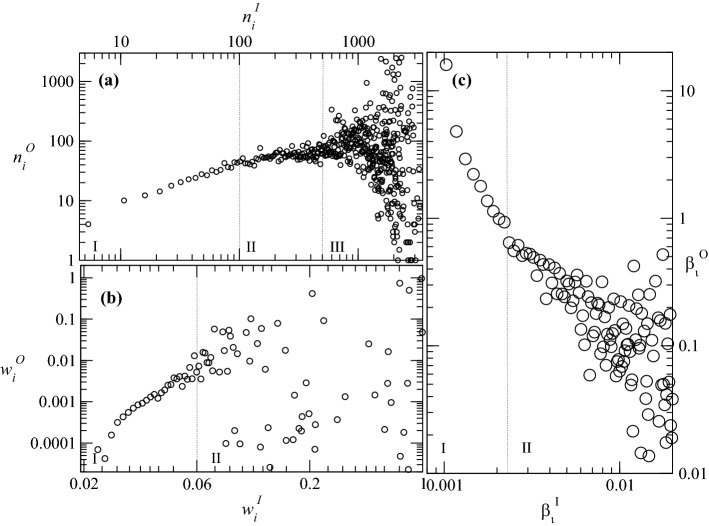
Comparing activity with impact and influencing neighborhoods with influenced neighborhoods: (**a**) $n_i^{I}\times n_i^{O}$: those who influenced the most are those who are influenced the least; (**b**) $w_i^{I}\times w_i^{O}$: activity and impact are not strongly correlated (**c**) $\beta _i^{I}=w_i^{I}/n_i^{I}\times \beta _i^{O}=w_i^{O}/n_i^{O}$: how much is the “influenced” level of *i* correlated with its respective “influencing” level?.

Figure 5a shows the average size of a user’s influenced neighborhood ($n_{i}^{O}$) over all users having an influencing neighborhood ($n_{i}^{I}$) of the same size in a log-log plot. The plot is visibly divided into three regions. Region I shows that the in-degree increases linearly with the out-degree for users *i* having $n_{i}^{I} < 100$. In other words, the number of people who influence a certain user *i* increases with the number of users that are influenced by *i*. The assumption that this region contains the group of occasional or “normal” users seems reasonable. Region II still shows a linear increase. In contrast to Region I however, the smaller slope indicates that users who are influenced by more than 300 but less than 700 people influence fewer users than they are influenced by. Region III differs from the other two in that it is divided into the influenced but not influential users in the lower right corner and the highly influential users in the upper right. Naturally, this group is the most impactful for the spread of information, i.e., though with a noisy value of $n_i^O$ all the users in this region are characterized by a large $n_i^I$. To note that this kind of asymmetry between in- and out-degrees of each user is a well known fact in directed social networks^31^.

Figure 5b shows the relationship between activity and impact rather than the neighborhoods. Like Fig. 5a, it is a log-log plot between $w_{i}^{O}$ and $w_{i}^{I}$. This plot is divided into two regions. Region I reflects the results of Fig. 4a and shows a linear correlation between a user’s activity and influence. This indicated that up to a certain degree one can become more influential by being more active. However, this is true until reaching a certain degree of activity. On the other hand, in Region II, no structure can be discerned, meaning there is no strong correlation between a user’s activity and impact.

To filter out the influence of the size of (influencing or influenced) neighborhoods, we introduce additionally two other measures, namely

6a $$\begin{aligned} \beta _{i}^{I}&{:}{=}&\frac{w_{i}^{I}}{n_{i}^{I}} \, , \end{aligned}$$

6b $$\begin{aligned} \beta _{i}^{O}&{:}{=}&\frac{w_{i}^{O}}{n_{i}^{O}} \, , \end{aligned}$$

which we interpret as the average impact (resp. activity) of user *i* to (resp. from) each one of his outgoing (resp. incoming) neighbors. In Fig. 5c, we show the average impact as a function of the average activity, uncovering an approximate inverse relation between both properties. The accounts that, on average, influence many people are themselves influenced by fewer people. Such individuals are often referred to as opinion leaders or trendsetters. In Region I, one can observe that the users that are, on average, not intensely influenced by their neighbors influence others stronger. This behaviour seems plausible since being influenced intensively by many neighbors requires time to consume and process the content, and the majority of users are not very active (see Fig. 5). Users in Region I seem to maintain a few but very strong connections. It should be taken into account that the presented weighting function normalizes over the connections to the respective neighbors. So users who often share content from few other users achieve high averages. Region II shows that this trend continues with increased variance.

**Figure 6:**
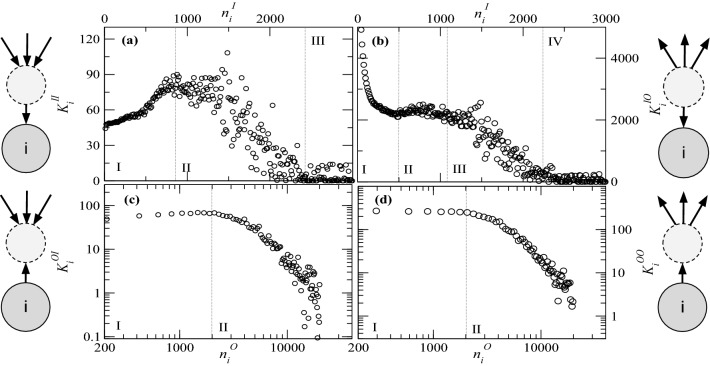
Relation between first and second neighborhoods, identifying regions with different user behaviors. (**a**) $K_i^{(II)} \times n_i^{(I)}$: how is the size of the influencing neighborhood of neighbors of a certain user *i* correlated with the size of their influencing neighborhood? There is a critical size beyond which disassortativity is observed. (**b**) $K_i^{(IO)} \times n_i^{(I)}$: how is the size of the influenced neighborhood of user *i* correlated with the size of its neighbors influencing neighborhood? Here one observes three regions: (I) one with few influencing neighbors and a lot of influenced neighbors (the “stars” of Twitter); (II) one “normal” region, where there is a typical value of the influenced neighborhood; and (III) a region with many influencing neighbors, who basically do not influence anyone. (**c**) $K_i^{(OI)} \times n_i^{(O)}$: how much are my neighbors influenced by others? Here one observes complete disassortative. (**d**) $K_i^{(OO)} \times n_i^{(O)}$: How is the size of the influenced neighborhood of neighbors of user *i* correlated with the size of their influenced neighborhood? Similar behavior as in (**c**).

### The second neighborhood: correlation between the activity and impact of a node with the activity and impact of its neighborhood

Based on the preceding definitions, it is now possible to define additional properties which connect the first two neighborhoods of a user *i*. In the following we investigate how influencing neighbourhood $n_{i}^{I}$ behaves to its influencing neighborhood by dividing the sum of *i*’s incoming neighbours’ in-degrees by *i*’s in-degree (see $K_{i}^{II}$ in Eq. 7a). Analogous to $K_{i}^{II}$ we define $K_{i}^{IO}$, $K_{i}^{OO}$ and $K_{i}^{OI}$ as follows.

7a $$\begin{aligned} K_{i}^{II}&{:}{=}&\frac{1}{n_i^{I}}\sum _{m \in {\mathcal {N}}_{i}^{I}}^{} {n_{m}^{I}} \, , \end{aligned}$$

7b $$\begin{aligned} K_{i}^{IO}&{:}{=}&\frac{1}{n_i^{I}} \sum _{m \in {\mathcal {N}}_{i}^{I}}^{} {n_{m}^{O}} \, , \end{aligned}$$

7c $$\begin{aligned} K_{i}^{OO}&{:}{=}&\frac{1}{n_i^{O}}\sum _{m \in {\mathcal {N}}_{i}^{O}}^{} {n_{m}^{O}} \, , \end{aligned}$$

7d $$\begin{aligned} K_{i}^{OI}&{:}{=}&\frac{1}{n_i^{O}} \sum _{m \in {\mathcal {N}}_{i}^{O}}^{} {n_{m}^{I}} \, . \end{aligned}$$

Here, (I) indicates the influencing and (O) the influenced neighborhood size of *i*’s influencing (I) or influenced (O) neighbor.

Figure 6a shows $K_i^{(II)} \times n_i^{(I)}$ or the average size of the neighbourhood that influences each of the users that influences *i*. In the first region one can observe that with every user that *i* retweets the average number of users that *i*’s influencer retweet grows linear. This is true until 800 influencer. In Region II, a drop in the curve can be observed. With each new influence of *i*, only users who are less influenced are added. The more one is influenced, the more difficult it is to be influenced by people who are influenced by as many or even more users. Finally, the curve in the third region is constant. Users who have been influenced by more than 2000 other users gain relatively few users who are very influenced.

Figure 6b depicts $K_i^{(IO)} \times n_i^{(I)}$ or the average number of users that were influenced by a user that influenced *i*. This plot can also be divided into four regions. In Region I, up to an in-degree of about 300, users are mainly influenced by influential users. One possible interpretation is that users who are not very active usually follow the stars and share their content, if at all. In Region I, it is also noticeable that the extremely large number of users (compared with Fig. 6a) who have only shared the content of a few users share the content of users who have an extremely high level of influence. The plot follows a power law in the first region. An appropriate label for users in Region I would be “The Fans”. In Region II, the curve flattens out and forms a plateau between users influenced by at least 300 but not more than 1000 other users. For users in this group, they are largely linked to users who are influential to the same extent that they are influenced. This seems especially interesting when comparing with Region I in Fig. 6a. here the opposite behavior is indicated for the average number of influencers per influencer of *i*. Therefore, the number of those who influence someone who influences *i* falls (in the same region), while the number of those who are influenced by those who influence *i* stagnates with the degree of influence. Region IV in b and Region III in a show again the same behavior.

Figure 6c shows the correlation between the out-degree of a user *i* and the average in-degree of all those who are influenced by *i*. Or in other words, the mapping shows for each user *k* who is influenced by *i*, from how many other users *k* is influenced (on average). This illustration can also be divided into two regions. In Region I, we observe the group of users who influenced less than 3000 users, and in the second region, we observe the group of users with more than 3000 users influenced.

Figure 6d shows the correlation between the out-degree of user *i* and the average out-degree of all those influenced by *i*. In other words, the plot shows for each user *k* who is influenced by *i*, how many other users influence *k* on average. A similar behavior to Fig. 6c is observed: two regions are also identified. In the first region, we find the user group that influences up to 2000 different users. Here, the average number of users that a neighbor influences sticks to about a hundred users, indicating that this group’s users tend to have influenced similar size neighborhoods. Moreover, users with influenced neighborhoods larger than $n_i^O\simeq 2000$ show disassortativity, typical of famous individuals (stars): the larger their influenced neighborhood, the smaller the influenced neighborhood of their neighbors is.

### Clustering coefficient and global measures of the network topology

As the final step of our topological analysis of the interaction network behind Twitter, we address three additional quantities. One is the local clustering coefficient while the other two are global measures: the shortest path length, $\ell _{ij}$, joining two users *i* and *j*, and the betweenness centrality $b_{i}$ of each user *i*.

**Figure 7:**
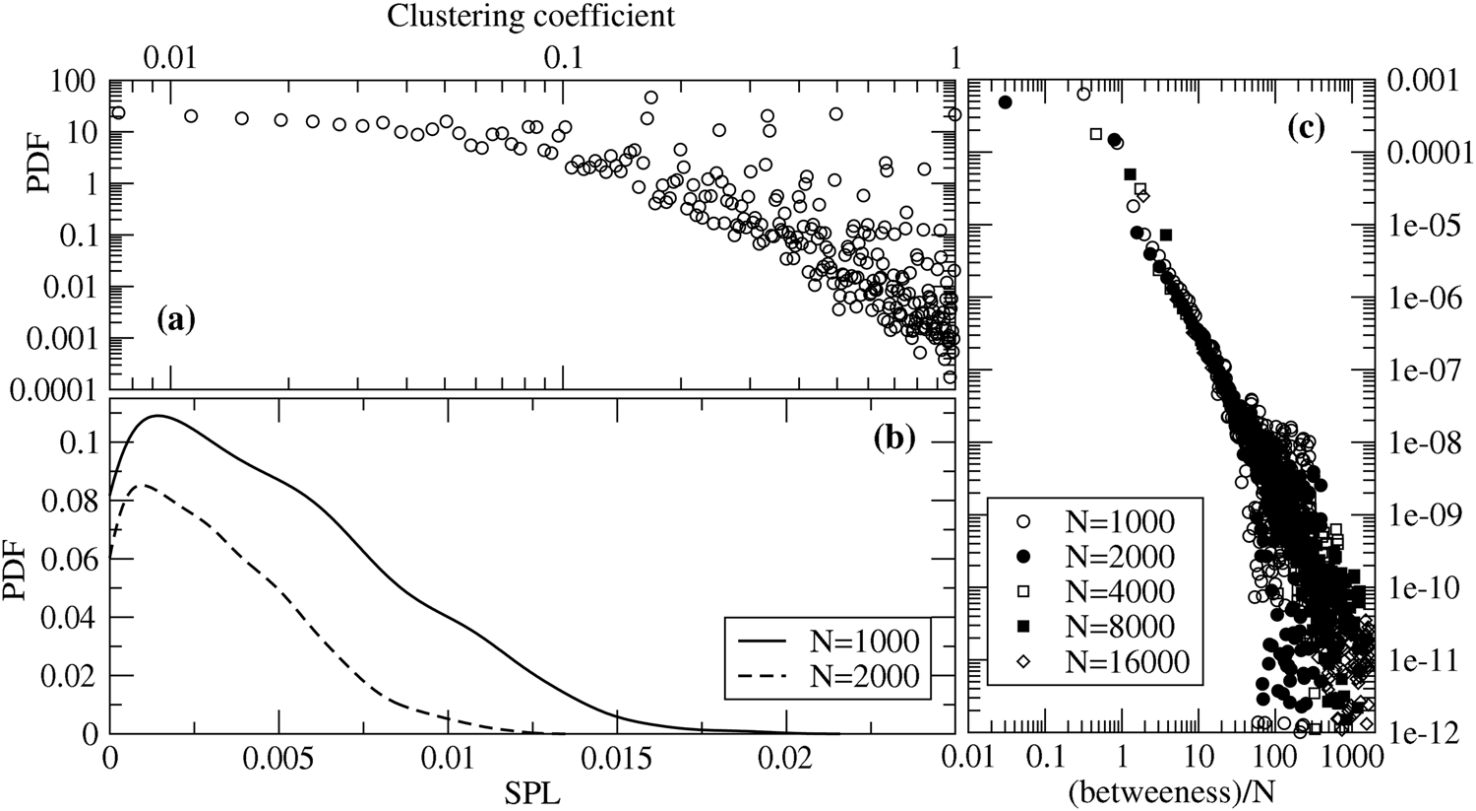
(**a**) Clustering coefficient spectrum of the derived network, (**b**) the respective shortest path length (SPL) spectrum and (**c**) betweenness centrality. While SPL, weighted by the values of $c_{ij}$, shows a unimodal spectrum, a mode at low values, and then an approximately linear decrease, both the clustering coefficient and the betweenness seem to have a polynomial decay. For performance reasons, only smaller sets of *N* nodes sampled from the main component were selected. As one observes, similar behavior is observed for different sizes of the sampled set of nodes, evidencing that results in this figure are representative of the entire network. We chose a random node from the largest component to create the subgraphs and performed a (directed) breadth first search from this node.

The clustering coefficient, $C_l (i)$, of a user *i* is the fraction of existing edges among its neighbors among all possible edges they may have. If one observes *m* of such edges, then

8 $$\begin{aligned} C_l(i) = \frac{2m}{n_i^A (1-n_i^A)} \, , \end{aligned}$$

where incoming and outgoing connections were considered as single undirected edges. As shown in Fig. 7a, one observes a slow decay in the number of nodes with a low clustering coefficient, between 1 and 10%, followed by an approximatelly polynomial decay.

The shortest path length $\ell _{ij}$ is defined as the shortest weighted path joining two different users, *i* and *j*. As shown in Fig. 7b, the spectrum of different $\ell _{ij}$ is broad and approximately unimodal.

The betweenness measures the level of “importance” of a node in the network by computing the amount of shortest paths crossing it. The distribution of node betweenness is plotted in Fig. 7c.

Note that, because of the network’s size and the resulting runtime for computing the shortest paths, we performed a selection of the network that we present in the course of this work. First, we extract the main component. Second, we perform 10,000 breath first searches (BFS) among a subset of the main component. For each of the runs we then increase the number of nodes picked up by an BFS run to later perform the shortest path analysis. Both Fig. 7b,c show that this method works because there is convergence in the respective plots, and the result retains its functional shape.

## Discussion and conclusions

We have introduced a framework to uncover a directed weighted network of connections based on tweet-retweet action from the German Twitter database. We used approx. 1 billion statuses (530 million tweets, 300 million retweets, and 170), building a network with approx. 120 million estimated weighted acquaintances among 70 million users where 30 million of them have a weighted interaction score ($c_{ij} > 0$).

Such a network can now be taken as a framework for investigating the social phenomena, completing it with additional information. The resulting scores constitute a dense representation of acquaintances for all pairs of users. Due to the size of typical social networks, such a representation is only practical for communities of interest in studying social phenomena, such as the spread of misinformation or hate speech. However, within such communities, the likely spread of information can easily be derived from the $c_{ij}$-scores, which is not possible from the social network’s link structure alone. For larger communities, it will be necessary to sparsify the representation by omitting negative scores from the representation.

The time window chosen for deriving the German Twitter network of acquaintances was before Covid-19 pandemic onset. The derived graph reflects German interactions almost in its entirety. Compared to other works we have not chosen to use the Twitter search API, choosing instead data from the user timelines and from the cummulative tweet dynamics directly. Still, one interesting question about how to detect the onset of pandemics in this twitter network. A possibility would be to repeat the same procedure as described in our manuscript for other specific time windows, just before and just after the pandemic onset.

While the network is build from the German Twiter database, it does not dependend on the content of the tweets and can, therefore, be applied to any Twitter dataset or to similar social networks. One interesting question is to investigate up to which extent the topological features reported here for the German Twitter subset prevail in other Twitter databases.

The derived network can now be used as a testbed to explore different social phenomena such as: how fast does news spread through the Twitter network? How are the dynamic features of news spreading in the Twitter network related to the topological properties around the users retweeting them? These and other questions will be addressed in future work.

### Ethical approval

All the procedures were performed in accordance with the relevant guidelines and regulations. This project has been ethical approved by the Oslo Metropolitan University (approval number 21/11732).
